# An analysis of epidemiological characteristics of microvascular complications and comorbidities among type 1 diabetes patients

**DOI:** 10.3389/abp.2025.14569

**Published:** 2025-05-22

**Authors:** Małgorzata Lewicka, Irmina Korzeniowska-Dyl, Dariusz Moczulski, Agnieszka Woźniak–Kosek, Magdalena Zawadzka, Gabriela Henrykowska

**Affiliations:** ^1^ Epidemiology and Public Health Department, Medical University of Lodz, Lodz, Poland; ^2^ Department of Internal Medicine and Nephrodiabetology, University Clinical Hospital No. 2 of the Medical University of Lodz, Lodz, Poland; ^3^ Department of Laboratory Diagnostics, Military Institute of Medicine – NationaI Research Institute, Warsaw, Poland; ^4^ Department of Hygiene and Epidemiology, Military Institute of Hygiene and Epidemiology, Warsaw, Poland

**Keywords:** complications, diabetic retinopathy, diabetic neuropathy, type 1 diabetes, comorbidities

## Abstract

**Purpose:**

Epidemiological analysis of medical data of patients with type 1 diabetes (T1DM) and disease complications treated in hospital.

**Methods:**

A retrospective, cross-sectional study was conducted on records from 306 patients with type 1 diabetes (180 men and 126 women). The study analyzed demographic, clinical, and biological data, focusing on associations between Hashimoto’s disease, neuropathy, and retinopathy using k-means clustering.

**Results:**

Hypertension was present in 28.8%, Hashimoto’s disease in 17.6%, retinopathy in 17.6%, neuropathy in 8.2%. Multivariate logistic regression showed that the chance of retinopathy more than doubles with the coexistence of hypertension (OR 2.096, 95% Cl: 1.035–4.248) and this chance increases by 4.5% with each year of age compared to the previous year (OR 1.045, 95% Cl: 1.011–1.080). The risk of neuropathy increases by 10.8% with each year since diabetes diagnosis compared to the previous year (OR = 1.108, 95% Cl: 1.062–1.156) and the chance of this disease rises by 17.6% with each year of diabetes duration compared to the previous year (OR 1.176, 95% Cl: 1.092–1.267). Clustering was strongest in patients without comorbidities (66.3%). Only 2.3% had Hashimoto’s disease and retinopathy, 3.59% had retinopathy and neuropathy, and just 1.3% had all three conditions.

**Conclusion:**

Patient age, duration of diabetes, and the presence of hypertension are key risk factors for diabetes-related complication.

## Introduction

Type 1 diabetes mellitus (T1DM) is a chronic disease characterized by abnormal metabolism and hyperglycemia due to insulin deficiency. It is caused by the immune system targeting islet cells and attacking and destroying pancreatic beta cells, resulting in a rapid decrease in insulin production. Symptoms include polyuria, polydipsia, vision disorders and weight loss (Shojaeian and Mehri-Ghahfarrokhi, 2018).

T1DM is often linked to diminished quality of life, severe long-term complications, decreased life expectancy, and significant financial burdens for both individuals and healthcare systems.

Despite advances in prevention and treatment, T1DM and its complications remain a significant public health problem worldwide. The results of meta-analysis showed that the incidence of type 1 diabetes in continental subgroups (Asia, Africa, Europe, and America) was 15 per 100,000, 8 per 100,000, 15 per 100, 000 and 20 per 100, 000, respectively (Mobasseri et al., 2020).

In 2021, there were approximately 8.4 million individuals worldwide living with type 1 diabetes. Among them, 1.5 million (18%) were under the age of 20, 5.4 million (64%) were between the ages of 20 and 59, and 1.6 million (19%) were aged 60 years or older. During that year, about 0.5 million new cases of T1DM were diagnosed, with the median age of onset being 29 years. Around 35,000 individuals who had not yet been diagnosed with the condition passed away within a year of experiencing symptoms. Of the total number of people affected by type 1 diabetes, approximately 1.8 million lived in low-income and lower-middle-income countries (Gregory et al., 2022).

The life expectancy of a 10-year-old diagnosed with T1DM in 2021 varied significantly based on the economic conditions of the region. The mean remaining lifespan ranged from around 13 years in low-income countries, falling to even eight years in the Central African Republic or Somalia to 65 years in high-income countries. In Poland the remaining life expectancy of a 10-year-old was 58 years (55–62 years) (Gregory et al., 2022).

While genetic susceptibility plays a critical role in the onset of type 1 diabetes, environmental factors have a more substantial impact on the progression from islet autoimmunity to the full-fledged disease. This is partly due to improved living standards, which have led to reduced exposure to microorganisms, potentially increasing the risk of autoimmunity (Forouhi and Wareham, 2014). However, acute fulminant diabetes, referred to as type 1b diabetes, has been documented following infection with Mumps, Coxsackie B3 and B4, Rubella, and Influenza B infection (Boddu et al., 2020).

Numerous studies have suggested that the risk of T1DM may be elevated by viral infections, particularly respiratory or enterovirus infections, as well as by Vitamin D deficiency (Lönnrot et al., 2017). Additionally, perinatal factors such as maternal age, a history of preeclampsia, and neonatal jaundice have also been associated with an increased risk. Interestingly, it has been observed that low birth weight is associated with a reduced risk of developing T1DM, whereas a high birth weight and lower gestational age at birth may increase it (Abbasi et al., 2017; Goldarce, 2018)., It is important to note that none of these associations have been definitively confirmed, and some have been contradicted by other research, which justifies the need for further research into the etiology of T1DM.

Araszkiewicz indicate that currently, there is no effective method of preventing type 1 diabetes, neither in the general population nor among people at risk (Araszkiewicz et al., 2023a). However, Kawasaki published that prevention is possible: primary prevention based on preventing seroconversion to one or more autoantibodies, including glutamic acid decarboxylase (GAD), anti-insulin, insulinoma-associated antigen 2 (IA2) and zinc-transporter 8, in those genetically at risk, and secondary prevention based on preventing loss of and damage to the beta-cells in individuals with autoimmunity/autoantibodies (Kawasaki, 2023). It should be noted that as of now, there are no certain methods to prevent type 1 diabetes, and most research is still in the experimental stage. Most efforts are focused on tertiary prevention, that is, measures to delay or limit the progression of the disease once it is detected early, and to prevent complications associated with the disease.

Chronic complications are widespread in both type 1 and type 2 diabetes patients and can lead to considerable illness and loss of life. These can be broadly categorized into two groups: microvascular and macrovascular, with the former being notably more prevalent. Microvascular complications encompass conditions like neuropathy, nephropathy, and retinopathy, while macrovascular complications include cardiovascular diseases, strokes, and peripheral artery diseases (PAD). Diabetic foot syndrome, a condition where foot ulcers are present alongside neuropathy, PAD, and infection, is a major contributor to lower limb amputation (Tuttolomondo et al., 2015).

To avoid the variety of potential complications and comorbidities, modern diabetes care must take into account the individual needs of patients. In Poland, treatment consists of outpatient care, primary healthcare and specialized hospital care. The latter is required primarily for acute complications (severe recurrent hyperglycemia or hypoglycemia, acidosis, diabetic comas), exacerbation of chronic complications or modification of the treatment regimen. In order to improve tertiary prevention for T1DM patients, a coordinated care system in primary healthcare was introduced in 2022 (Ministry of health, 2022). Under coordinated care patients are given the opportunity to undergo an annual comprehensive health review, which allows for the creation of a personalized medical care plan, called a “roadmap for the coming year.” Patients also gain easier access to diagnostic tests that help detect possible complications, these can include monitoring kidney function and lower extremity vascular testing to assess diabetic foot risks. In addition, diabetic patients receive six educational sessions and three dietary consultations annually. The primary care physician has the option of consulting a diabetologist or referring the patient to a specialized outpatient clinic, especially in situations where there are doubts about the patient’s health or there is a need for further treatment.

It is also worth noting that the eligibility for coordinated care has recently been extended to include patients under the age of 18 in primary care, effective 1 July 2023.

Patient care emphasizes the importance of dietary and therapeutic education, which should be carried out at every visit, as well as access to a nutritionist, psychologist and specialized consultations.

The primary objective of this study was to determine the incidence and epidemiological characteristics of diabetes-related complications, particularly among middle-aged individuals (the average age of patients was 38 years). This research highlights the ongoing healthcare needs of this demographic, providing a detailed epidemiological analysis of type 1 diabetes complications. The study’s approach allows for a deeper understanding of the prevalence of these complications and the factors that may influence them.

Furthermore, few studies have explored individuals who remain free of complications, and the patterns of co-occurrence and interactions among all three microvascular complications in T1DM have yet to be fully understood. In this study, we aim to describe the extent of clustering among microvascular complications.

By doing so, it offers valuable insights that can enhance prevention and treatment strategies, inform the adjustment of healthcare programs, and increase public awareness of the importance of early detection and intervention in diabetes complications.

Despite diabetes complications being well-documented for years, systematic studies are needed to assess the progress in medical care for these patients, as well as advancements in primary and secondary prevention.

## Material and methods

The study was conducted at the Department of Internal Medicine and Nephrodiabetology of the University Clinical Hospital No. 2 of the Medical University of Lodz, Poland (former name: Military Medical Academy Memorial Teaching Hospital, Medical University of Lodz–Central Veterans’ Hospital). Patients were admitted as inpatients to monitor the progress of the disease and its complications, and to address any abnormalities. The duration of hospitalization was brief, lasting 3–4 days, with parameters measured once during this period.

A retrospective analysis of the medical records of 306 patients (180 men and 126 women) hospitalized over the past 7 years was conducted.

Exclusion criteria included the lack of confirmed diagnosis of type 1 diabetes—for example, in cases with an ambiguous diagnosis or insufficient data to distinguish it from other forms of diabetes such as LADA or MODY; incomplete medical records, including missing key information necessary for analysis such as laboratory results, demographic data, or a complete medical history; and a short disease duration—less than 1 year since diagnosis. This study was approved by the Ethics Committee of Medical University of Lodz (no. RNN/260/18/KE).

The following data were analyzed: age, sex, age at diabetes diagnosis, duration of disease, Body Mass Index (BMI), smoking, family history, hypertension (HA), Hashimoto’s disease, retinopathy, neuropathy.; The following parameters were analyzed: C-reactive protein (CRP), Glomerular Filtration Rate (GFR), Uric Acid (UA), Urinary Albumin-to-Creatinine Ratio (UACR), Serum Creatinine (CREAT), Thyroid-Stimulating Hormone (TSH), Free Triiodothyronine (FT3), Free Thyroxine (FT4), Thyroid Peroxidase Antibodies (TPO antibodies), Hemoglobin A1c (HbA1c), Cholesterol (Chol), Triglycerides, LDL (Low-Density Lipoprotein), HDL (High-Density Lipoprotein).

The k-means clustering method was performed on three binary variables: Hashimoto’s disease, neuropathy and retinopathy and used it to calculate expected counts of persons with no complications, with each separate complication, with each complication pair and with all three microvascular complications, under the null hypothesis of no association between the three microvascular complications.

Then, a separate analysis of sixteen clusters was performed, including hypertension. Analyses by age group were abandoned due to too few cases of co-occurrence.

The data considered in this study were selected based on their prevalence in the sample population and their potential statistical significance. Macrovascular complications were excluded from the analysis due to their low incidence.

### Statistical analysis

The mean (M), median (Mdn), minimum-maximum (min-max) and standard deviation (SD) were calculated for the patient data. As the parameters had a non-normal distribution, they were analyzed using the Mann-Whitney test. The impact of 23 quantitative and qualitative predictors on the occurrence of retinopathy, neuropathy and Hashimoto’ disease was determined by logistic regression. Any predictors with an LR (likelihood ratio) test result of p < 0.05 were removed from further analysis (age, age at diabetes diagnosis, years of diabetes, HA, TSH). To assess the goodness of fit for the logistic regression models, a Hosmer-Lemeshow test was conducted. A p-value of <0.05 was considered statistically significant. To present the results more precisely, exact *p*-values were provided where available. However, when the *p*-value was extremely low (e.g., below 0.0001), it was reported as P < 0.05 for clarity. The k-means clustering method was used to analyze the associations between Hashimoto disease, neuropathy, and retinopathy among patients with T1DM.

The results were analyzed using the Statistica 13.3 Statistical Package.

## Results

Of the 306 participants analyzed, 180 (58.8%) were men and 126 were women (41.2%). Seventeen patients were rejected due to having more than 50% missing data. The mean age of the participants was 37.6 years (range 25–73). Due to the wide age range, cluster analysis using the k-means method was applied. The study group was divided into three clusters: the first with a mean age of 30 years, the second with a mean age of 41 years, and the third with a mean age of 61 years (Table 1).

**TABLE 1:** Age clusters based on k-means analysis.

Age clusters based on k-means analysis (total number of cases: 306)				
Parameter	Age 52–73	Age 36–51	Age 25–35	Total
Minimum	52.0	36.0	25.0	25.0
Maximum	73.0	51.0	35.0	73.0
Mean	61.2	41	30.2	37.6
SD	5.6	3.5	2.7	10.0
Persentage (%)	10.13%	39.54%	50.33%	100%

By sex, the mean ages were 38.3 years (range 25–68) for women and 37.1 years (range 25–73) for men. The mean age of diabetes diagnosis was 15.2 years (range 0–65); by sex were 14.9 years (range 0–65) for men and 15.5 years (range 1–57) for women. The mean duration of diabetes was 22.3, ranged from 1 to 43 years (for men 22.2, range 1–43 years; for women 22.5, range 6–39 years).

The mean BMI was 25.77 kg/m^2^ (26.13 for men; 25.25 kg/m^2^ for women). BMI ranges from 17.71 to 41.36 kg/m^2^ (for men 18.08–41.36; for women 17.71–36.25 kg/m^2^). 34.80% (n = 79) of the patients were overweight, and an additional 16.74% (n = 38) fell into various categories of obesity (BMI class 1%–12.78%, n = 29; class 2%–3.52%, n = 8; class 3%–0.44%, n = 1). This means that over half of the group (51.98%) were above the normal weight range. 2.2% (n = 5) of patients were underweight, and 46.26% (n = 105) had a normal BMI. The remaining parameters listed in the Materials and Methods are presented in Tables 2–4 (mean, median, min-max, SD).

**TABLE 2:** Concentration of CRP, GFR, UA, UACR, CREAT depending on sex and total outcome.

Parameter		Female (n = 126)	Male (n = 180)	Total (n = 306)
CRP [mg/L]	M	3.4	2.3	2.8
	Mdn	2.2	1.2	1.4
	Min-max	0.2–15.5	0.1–22.8	0.1–22.8
	SD	3.3	3.2	3.3
GFR [mL/min/1.73m^2^]	M	103.5	92	96.7
	Mdn	105.4	97.7	101.2
	Min-max	64.7–138.5	38.7–131.6	38.7–138.5
	SD	15.6	20	19.1
UA [µmol/L]	M	224.5	291.3	263.8
	Mdn	214.3	290	271.7
	Min-max	148–375	144–565	144–565
	SD	41	42.7	53.3
UACR [mg/g]	M	8.5	10.4	9.6
	Mdn	5.6	3.7	4.3
	Min-max	1.1–53.1	0.2–500	0.2–500
	SD	8.5	44.7	34.7
CREAT [mg/dL]	M	0.7	0.9	0.8
	Mdn	0.3–1.1	0.9	0.8
	Min-max	0.6–1.3	0.6–1.3	0.3–1.3
	SD	0.1	0.1	0.2

**TABLE 3:** Concentration of CHOL, TG, LDL, HDL depending on gender and total outcome.

Parameter		Female (n = 126)	Male (n = 180)	Total (n = 306)
CHOL [mmol/L]	M	4.60	4.60	4.60
	Mdn	4.60	4.60	4.60
	Min-max	3–7.4	3.1–9.8	3–9.8
	SD	0.70	0.90	0.80
Triglyceride [mmol/L]	M	1.10	1.20	1.20
	Mdn	0.90	1.10	1.00
	Min-max	0.4–4.3	0.4–4.2	0.4–4.3
	SD	0.60	0.70	0.70
LDL [mmol/L]	M	2.60	2.70	2.70
	Mdn	2.60	2.60	2.60
	Min-max	1.2–4.8	1.3–5.7	1.2–5.7
	SD	0.60	0.70	0.70
HDL [mmol/L]	M	1.50	1.40	1.40
	Mdn	1.50	1.30	1.40
	Min-max	0.9–2.5	0.7–3.6	0.7–3.6
	SD	0.30	0.40	0.30

**TABLE 4:** Concentration of TSH, FT3, FT4, Anti-TPO, HbA1c depending on sex and total outcome.

Parameter		Female (n = 126)	Male (n = 180)	Total (n = 306)
TSH [µIU/mL]	M	2.3	2	2.1
	Mdn	1.8	1.8	1.8
	Min-max	0.07–9.3	0.01–13.3	0.01–13.3
	SD	1.7	1.4	1.6
FT3 [pmol/L]	M	4.5	5.2	4.9
	Mdn	4.5	5.2	4.9
	Min-max	2.9–6.8	3.2–9.3	2.9–9.3
	SD	0.5	0.6	0.7
FT4 [pmol/L]	M	15.4	15.5	15.4
	Mdn	15.2	15.6	15.4
	Min-max	8.9–27.5	9.2–21.9	8.9–27.5
	SD	2.4	2	2.2
Anti-TPO [IU/mL]	M	114.9	42.5	72.3
	Mdn	25.6	11.5	13.8
	Min-max	5–610	4–463.8	4–610
	SD	157	78.5	122.4
HbA1c [%]	M	7.5	7.4	7.7
	Mdn	7.8	7.6	7.4
	Min-max	5.7–14.5	5.2–13	5.2–14.5
	SD	1.6	1.3	1.4

In the study population, a total of 75 subjects (24.5%) had no family history of diabetes type 1. Also, 164 subjects (53.6%) had a family history of T1DM on the mother’s or father’s side, while 47.8% (n = 86) of men and 61.9% (n = 78) of women declaring the presence of diabetes in one of their parents. In addition, 67 patients (21.9%) had a family history of diabetes on the grandmother’s or grandfather’s side; this was more common among men (23.3%; n = 42) than women (19.8%; n = 25).

Among the subjects, 26.1% (n = 80) were smokers; of these 30% (n = 24) were women and 70% (n = 56) were men.

Among the 306 patients diagnosed with type 1 diabetes, hypertension was found to be present in 28.8% (n = 88): 19% (n = 17) women and 81% (n = 71) men. Among the entire group of patients diagnosed with type 1 diabetes Hashimoto’s disease was identified in 54 (17.6%) patients and was more prevalent among women, i.e. 63% of the cases (34 patients), than men, i.e. 36.5% (20 patients). Retinopathy was also detected in 54 patients, making up 17.6% of the total cases. The sex distribution for retinopathy was relatively balanced, with women comprising 44.4% (n = 24) and men 55.6% (n = 30). Neuropathy was found in 25 individuals, accounting for 8.2% of the total patient population. The incidence of neuropathy did not show a significant sex-based difference. Among patients with neuropathy, women made up 52% (13 patients), while men accounted for 48% (12 patients).

Incidence of complications:

The dependence of the Hashimoto’s disease, retinopathy, neuropathy on the variables studied is shown in the Tables 5–7.

**TABLE 5:** Dependence of Hashimoto’s disease on studied variables. Aggregate results.

Evidence of Hashimoto’s disease	Variable	Mean	Median	Min.	Max.	SD
No	Age	37.3	35	25	73	9.9
	Duration of diabetes	22.1	22	1	43	7.4
n = 252	BMI	25.77	25.37	17.71	41.36	3.9
	Age at diabetes diagnosis	15	13	0	65	10.7
YES	Age	38.9	38	25	65	10.4
	Duration of diabetes	23.2	21.5	11	41	8
n = 54	BMI	25.78	25.75	18.07	36.25	4.5
	Age at diabetes diagnosis	15.7	12	3	51	11.3

**TABLE 6:** Dependence of retinopathy on studied variables. Aggregate results.

Evidence of Retinopathy	Variable	Mean	Median	Min.	Max.	SD
No	Age	36.4	34	25	73	9.4
	Duration of diabetes	21.1	21	1	43	7
n = 252	BMI	25.5	25.33	17.71	37.81	3.7
	Age at diabetes diagnosis	15.2	13	0	65	10.8
Yes	Age	43.4	41	29	73	11
	Duration of diabetes	28.2	29	16	41	7
n = 54	BMI	27.03	26.13	19.22	41.36	4.7
	Age at diabetes diagnosis	15.02	13	1	57	11.2

**TABLE 7:** Dependence of neuropathy on studied variables. Aggregate results.

Evidence of Neuropathy	Variable	Mean	Median	Min.	Max.	SD
No	Age	36.4	34	25	73	8.7
	Duration of diabetes	21.9	21	1	41	7.3
n = 281	BMI	25.79	25.43	17.71	41.46	4
	Age at diabetes diagnosis	14.3	12	0	65	9.8
Yes	Age	51.3	51	26	73	13.4
	Duration of diabetes	26.6	27	12	43	8.8
n = 25	BMI	25.58	25.32	19.22	35.3	3.8
	Age at diabetes diagnosis	24.7	19	3	57	16.2

Regarding the predictive statistics, no statistically significant relationships were found between the duration of diabetes and the presence of Hashimoto’s disease (p = 0.576), between Hashimoto’s disease and BMI (p = 0.951), between the incidence of retinopathy and BMI size (p = 0.055), nor between the occurrence of neuropathy and size of BMI (p = 0.846).

**FIGURE 1:**
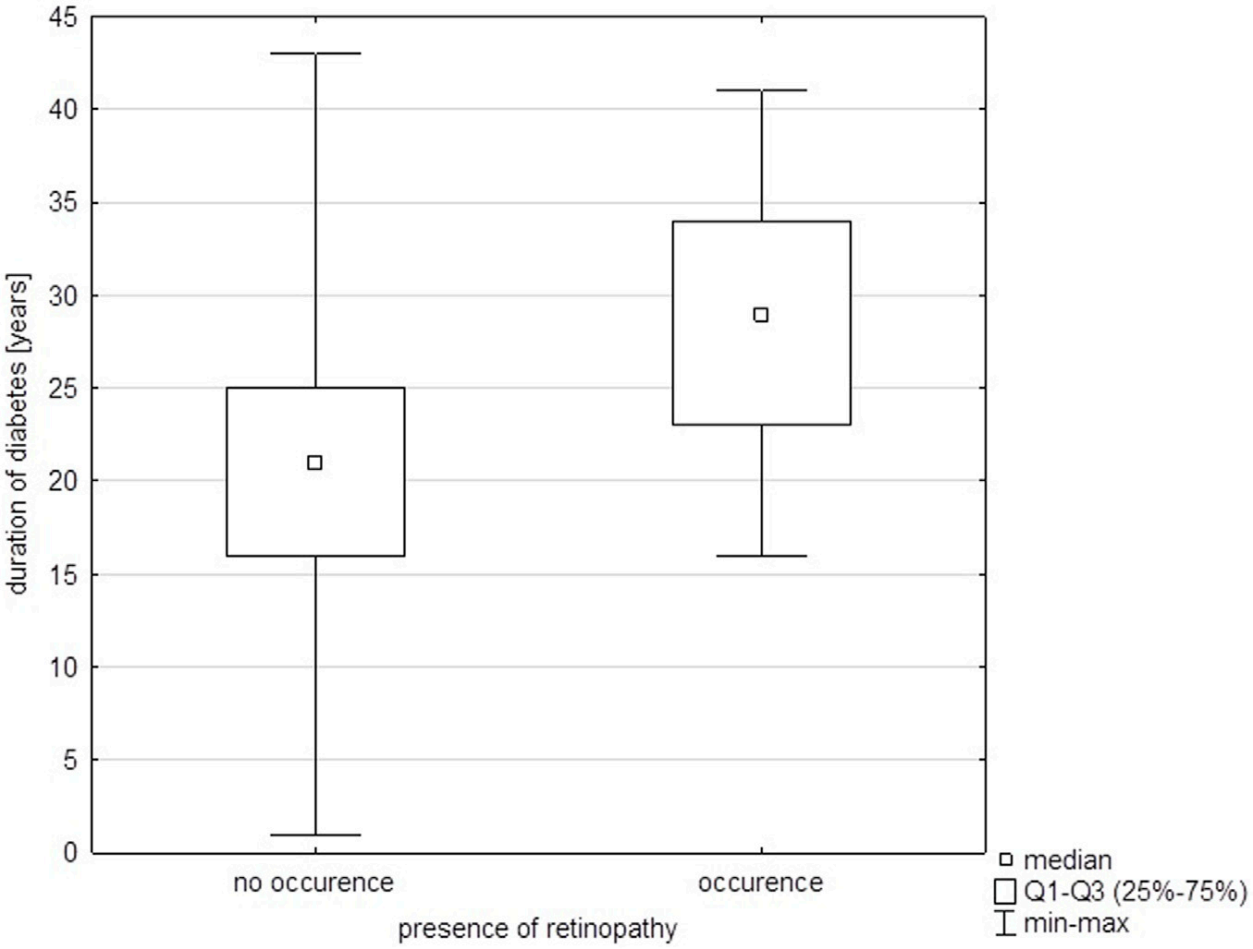
Duration of diabetes vs. presence of retinopathy.

Significant relationships were found between the duration of diabetes and the occurrence of retinopathy, with retinopathy occurring six times more frequently in individuals with a longer duration of diabetes (p < 0.05) (Figure 1).

**FIGURE 2:**
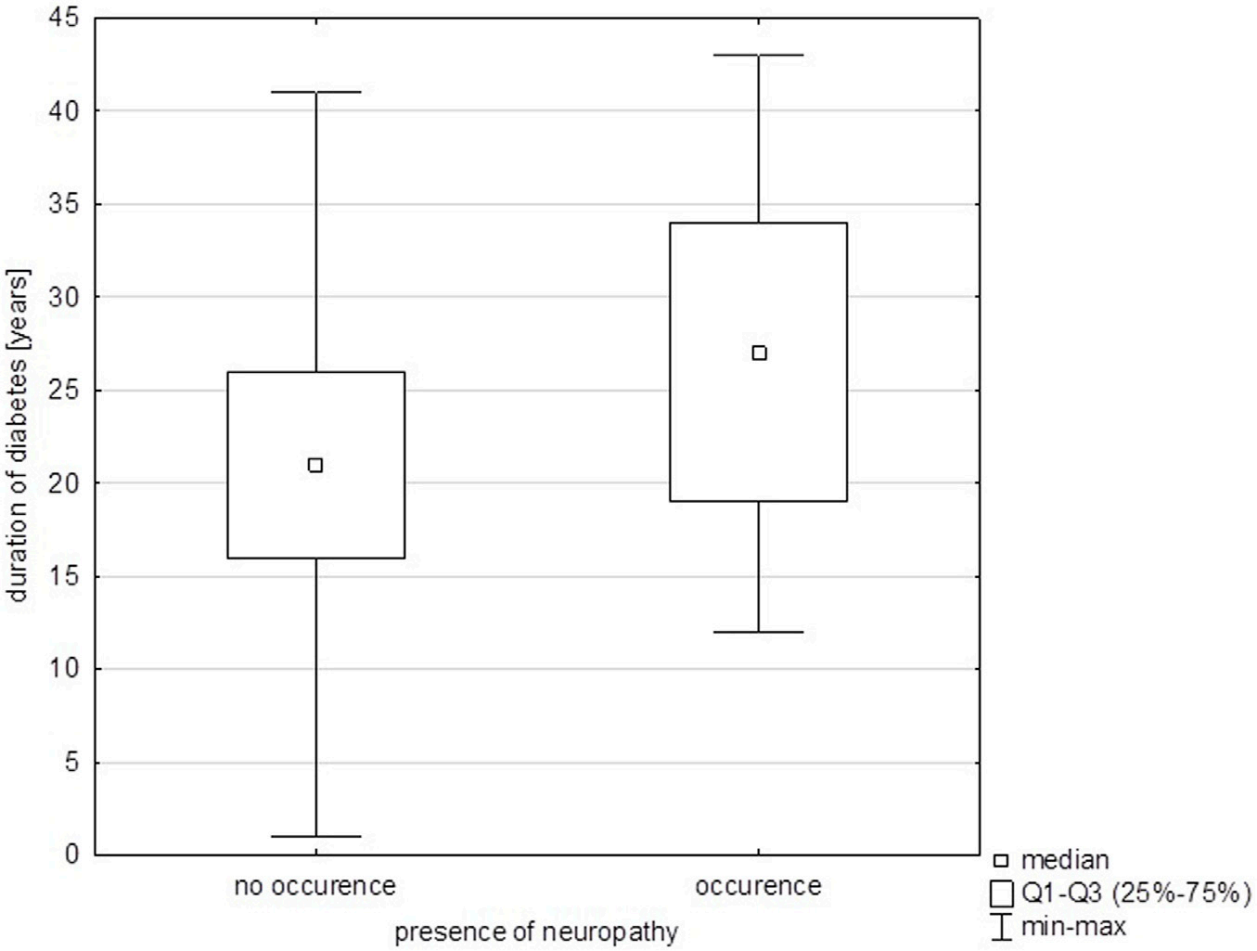
Duration of diabetes vs. presence of neuropathy.

Also, a statistically significant relationship was observed between the duration of diabetes and the occurrence of neuropathy with a 2.5 times higher risk of developing neuropathy associated with each year increase in the duration of diabetes (p = 0.01) (Figure 2).

**FIGURE 3:**
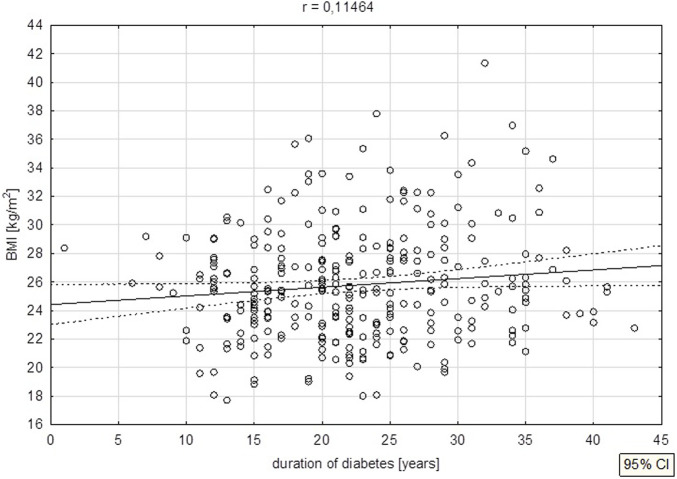
Duration of diabetes vs. BMI.

In addition, in univariate correlation the length of the duration of diabetes correlated positively with BMI (p = 0.045) (Figure 3).

**FIGURE 4:**
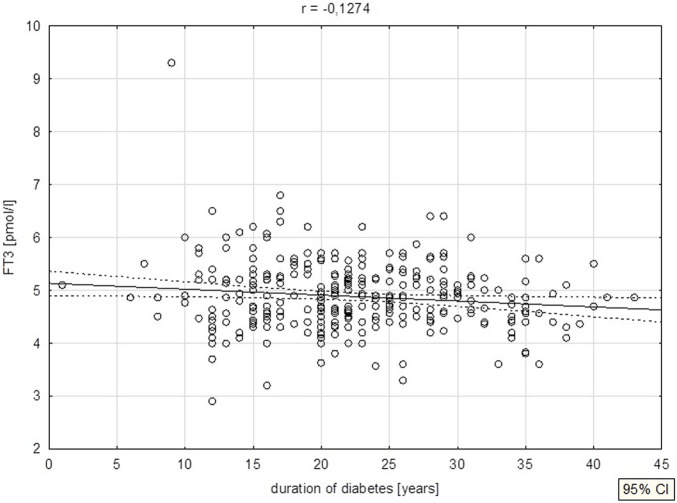
Duration of diabetes vs. FT3 levels.

**FIGURE 5:**
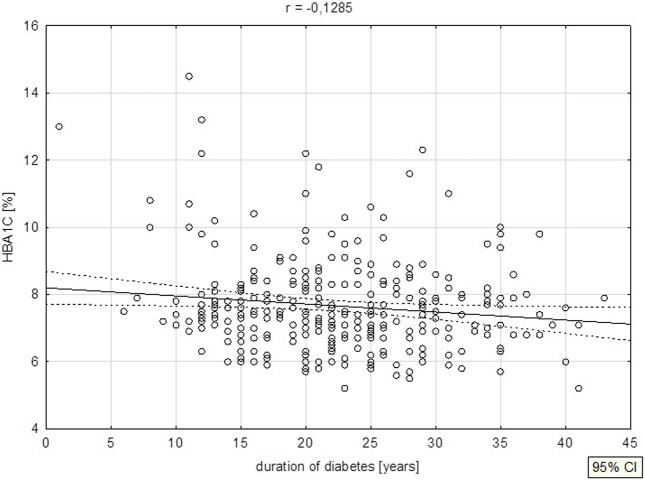
Duration of diabetes vs. HBA1C.

A negative correlation was noted between diabetes duration and FT3 levels (p = 0.026) (Figure 4), as well as HBA1C levels (p = 0.025) (Figure 5).

Positive correlations were found between BMI and the following parameters: CRP (p < 0.05) (Supplementary Figure S1); UA (p = 0.013) (Supplementary Figure S2); FT3 (p = 0.048) (Supplementary Figure S3); LDL (p < 0.05) (Supplementary Figure S4); TG (p < 0.05) (Supplementary Figure S5); CHOL (p < 0.05) (Supplementary Figure S6); and a negative correlation between BMI and HDL levels (p < 0.05) (Supplementary Figure S7).

Multivariate logistic regression was conducted to examine the impact of predictors on the occurrence of Hashimoto’s disease, retinopathy, and neuropathy.

The chances of Hashimoto’s disease occurrence increase by 2.2% with each unit increase in GFR (OR 1.002, 95% Cl: 1.002–1.043; p = 0.042) and by 0.6% with each unit increase in TPO antibodies (OR 1.006, 95% Cl: 1.003–1.008; p < 0.05), Table 8. The model demonstrated a moderate fit for the analyzed variable (AUC = 0.769; Hosmer-Lemeshow test).

**TABLE 8:** Multivariate logistic regression results for Hashimoto’s disease predictors.

Hashimoto’s disease predictor	Model evaluation	Standard error (SE)	Wald χ2	UCL 95%	LCL 95%	p	OR	OR CL –95	OR CL 95%
β 0	−4.618	2.091	4.876	−8.717	−0.519	0.027	0.010	0.000	0.595
GFR [mL/min/1.73m^2^]	0.022	0.010	4.605	0.002	0.042	0.032	1.002	1.002	1.043
TPO antibodies [lU/mL]	0.006	0.001	20.464	0.003	0.008	0.000	1.006	1.003	1.008

The chance of retinopathy disease occurrence more than doubles with the coexistence of hypertension (OR 2.096, 95% Cl: 1.035–4.248; p = 0.04) and increases by 4.5% with each year of age compared to the previous year (OR 1.045, 95% Cl: 1.011–1.080; p = 0.009). It also rises by 12.8% with each year of diabetes duration compared to the previous year (OR 1.128, 95% Cl: 1.077–1.182; p < 0.05), Table 9. The model demonstrated an adequate fit for the analyzed variable (AUC = 0.793; Hosmer-Lemeshow test).

**TABLE 9:** Multivariate logistic regression results for retinopathy predictors.

Retinopathy predictor	Model evaluation	Standard error (SE)	Wald χ2	UCL 95%	LCL 95%	p	OR	OR CL –95	OR CL 95%
β 0	−7.681	1.347	32.516	−10.322	−5.041	0.000	0.000	0.000	0.006
HA	0.740	0.360	4.220	0.034	1.447	0.040	2.096	1.035	4.248
Age	0.044	0.017	6.762	0.011	0.077	0.009	1.045	1.011	1.080
duration of disease	0.120	0.024	25.451	0.074	0.167	0.000	1.128	1.076	1.182

The chance of neuropathy disease occurrence increases by 10.8% with each year since diabetes diagnosis compared to the previous year (OR = 1.108, 95% Cl: 1.062–1.156; p < 0.05) and rises by 17.6% with each year of diabetes duration compared to the previous year (OR 1.176, 95% Cl: 1.092–1.267; p < 0.05). It was also increased by 23.2% with each unit increase in FT4 (OR 1.233, 95% Cl: 1.017–1.495; p = 0.033), Table 10. The model demonstrated a good fit for the analyzed variable (AUC = 0.862; Hosmer-Lemeshow test).

**TABLE 10:** Multivariate logistic regression results for neuropathy predictors.

Neuropathy predictor	Model evaluation	Standard error (SE)	Wald χ2	UCL 95%	LCL 95%	p	OR	OR CL –95	OR CL 95%
β 0	−11.409	2.337	23.827	−15.990	−6.828	0.000	0.000	0.000	0.001
age at diagnosis	0.103	0.021	22.794	0.060	0.145	0.000	1.108	1.062	1.156
duration of disease	0.162	0.038	18.366	0.088	0.237	0.000	1.176	1.092	1.267
FT4 [pmol/L]	0.209	0.098	4.542	0.017	0.402	0.033	1.233	1.017	1.495

Clustering of microvascular complications:

The k-means clustering method was used to analyze the associations between Hashimoto disease, neuropathy, and retinopathy among patients with T1DM. The majority of patients (66.3%) do not have any of the three disease, suggesting that most diabetic patients in this study do not suffer from coexisting diseases like Hashimoto, retinopathy, or neuropathy. Smaller groups of patients exhibit various combinations of these diseases, with the most common clusters including those with only Hashimoto (12.75%) or only retinopathy (10.46%). Only a few groups of patients suffer from more than one disease, such as Hashimoto and retinopathy (2.29%) or retinopathy and neuropathy (3.59%). The group where all three diseases coexist is very small (1.31%).

## Discussion

Our findings indicate that despite continual care for individuals with T1DM, complications frequently persist, and the quality of life for these patients remains suboptimal.

In general although most autoimmune diseases are more common in women. In our study results indicate a higher prevalence among male patients diagnosed with T1DM (180 men vs. 126 women). Other studies also indicate a greater risk of T1DM among male European patients 13 years of age or older (Gale and Gillespie, 2001; Harjutsalo et al., 2008) and a study conducted among children under 6 years old in Massachusetts, USA found T1DM in every three boys to every two girls (Quinn et al., 2006).

In 2022, 1.52 million individuals diagnosed with T1DM (17.0% of all T1DM patients) were under the age of 20, 5.56 million (64.0%) were between 20 and 59 years old, and 1.67 million (19.9%) were 60 years or older. In 2022, the total number of new T1DM diagnoses reached 530,000 across all age groups, among which 201,000 cases were identified in individuals younger than 20 years old (Ogle et al., 2022). In general, all populations show a steady increase in incidence with age up to about 10–15 years; recent data from Finland found the incidence in children aged 0–4 years to be almost as high as in those aged 10–14 years (Harjutsalo et al., 2013). The occurrence of T1DM in childhood follows a bimodal pattern, characterized by two distinct peaks: one between the ages of four to six and another during early puberty (10–14 years of age). Overall, approximately 45 percent of children develop symptoms before the age of 10. Some populations demonstrate a second increase in T1DM after the age of around 25–30 years (Levitsky and Misra, 2023).

T1DM has a strong genetic basis, identified through the HLA class II DRB1-DQB1 locus and several class I alleles (Cardinale et al., 2023). Our present data indicate that the incidence of T1DM is indeed influenced by familial relationships. In the study population, 164 subjects (53.6%) had T1DM in the family on the maternal or paternal side, with 47.8% (n = 86) of men and 61.9% (n = 78) of women declaring the presence of T1DM in one of their parents. In addition, 21.9%, n = 67 respondents (42 men and 25 women) had T1DM in the family on the side of their grandmother or grandfather.

Tillil et al. and Steck et al. found the likelihood of developing T1DM to be substantially higher among close relatives of individuals diagnosed with T1DM (Tillil and Köbberling, 1987; Steck et al., 2005). Moreover, a higher likelihood of developing T1DM has been recorded when a father is also affected compared to a mother (Turtinen et al., 2019). Indeed, the risk of developing T1DM is greater where a sibling (8%), father (5%), or mother (3%) also has T1DM (Pociot and Lernmark, 2016).

Individuals whose first degree relative has T1DM have a more than 15-fold increased risk of T1DM;, about 85% of those with a new diagnosis have no family history of T1DM (Ziegler et al., 2016).

Complications in type 1 diabetes are common and can have a range of causes. Many patients demonstrate quantitative and qualitative changes in the lipid profile. A significant role in the etiology of lipid disorders in T1DM is played by absolute insulin deficiency, resulting in the inhibition of lipogenesis and more intense lipolysis, resulting in increased levels of free fatty acids and triglycerides in the blood. In individuals with type 1 diabetes, the most common lipid phenotype involves low levels of HDL cholesterol and elevated LDL fractions. Even when hyperglycemia is controlled, individuals with T1DM still face a doubled risk of mortality (Dilworth et al., 2021). Hence, managing high blood sugar alone might not be enough to address cardiovascular risk and reduce mortality in T1DM. Dyslipidemia has been a long-considered factor contributing to cardiovascular disease risk in these individuals (Bebu et al., 2017; Miller et al., 2019).

In the presented study, the mean LDL concentration was 2.7 mmol/L (ranges 1.2–5.7 mmol/L). Normal LDL concentrations for individuals with diabetes are 1.0 mmol/L, 1.4 mmol/L, and 1.8 mmol/L, depending on cardiovascular risk (extreme, very high, high risk). The LDL concentration in young individuals under 35 years of age with type 1 diabetes, without chronic complications and other cardiovascular risk factors, should be 2.6 mmol/L (Araszkiewicz et al., 2023b).

As the results show, the average LDL-C was above normal. Hence, it is essential to determine the cardiovascular risk in each patients with T1DM, and to monitor and control LDL-C and non-HDL-C levels by adjusting the diet, encouraging physical activity, and in some cases initiating drug treatment (e.g., statins as first-line drugs) to lower the risk of cardiovascular disease. Proper glycemic control is very important for countering lipid disorders, especially hypertriglyceridemia.

Another major complications of type 1 diabetes is diabetic kidney disease (DKD), but its prognosis remains a challenge. A number of studies have suggested that early changes in glomerular filtration rate (GFR) and the presence of an elevated albumin-to-creatinine ratio (ACR) may be early indicators of diabetes-related renal problems in adolescents (Lovshin et al., 2018; Bjornstad et al., 2017). These findings underscore the importance of monitoring renal function and ACR levels as potential risk indicators for the development of diabetic nephropathy in individuals with T1DM. An ACR greater than or equal to 30 mg/g of creatinine is usually considered an indicator of the presence of pathological levels of albumin in urine (Martínez-Castelao et al., 2014). However, some guidelines and expert groups have lowered this limit to 17 mg/g for men and 25 mg/g for women (Scottish Intercollegiate Guidelines Network, 2008).

In the present study, the mean eGFR was 96.7 mL/min/1.73 m^2^ (Mdn: 101.2; ranges 38.7–138.5 mL/min/1.73 m^2^). Higher values were noted in women than in men (Mdn: 105.4 vs. 97.7 mL/min/1.73 m^2^). Although the mean ACR values were 9.6 mg/g, with a median of 4.3 mg/g (for men: M 10.4, Mdn 3.7; for women: M 8.5, Mdn 5.6 mg/g), staying within the normal range significant deviations from the norms were noted in some patients, with extreme values ranging from 0.2 to 500 mg/g (for men: 0.2–500; for women: 1.1–53.1 mg/g). Similarly, in men extremely low GFR values below the normal range were noted, i.e. 38.7 mL/min/1.73 m^2^.

Exposito et al. report that men with ACR values ≥17 mg/g and women with values ≥25 mg/g exhibited early signs of chronic kidney disease. The study found that 7% of men and 1.5% of women had this condition, i.e., a notably higher prevalence in men (Expósito et al., 2019).

Besides the genetic predisposing factors and environmental determinants, hyperglycemia, dyslipidemia, elevated blood pressure, and smoking have been highlighted as significant influencing the development and progression of kidney dysfunction in patients with T1DM (Pacilli et al., 2017; Feodoroff et al., 2016). Consequently, diabetic kidney disease pathology is complex and necessitates a comprehensive evaluation of modifiable risk factors.

The incidence of complications in patients, such as the occurrence of Hashimoto’s disease, retinopathy, and neuropathy, was also investigated.

Hashimoto thyroiditis is the most common autoimmune thyroid disease as well as the most often concomitant autoimmune disorder coexisting with TDM1.

Among the 306 patients diagnosed with type 1 diabetes, Hashimoto’s disease was identified in 54 of them, which accounts for 17.6% of the total. Notably, Hashimoto’s disease was more prevalent among women with type 1 diabetes, constituting 63% of the cases (n = 34), while men represented 36.5% (n = 20) of this group.

This observation has been supported by other researchers in the context of autoimmune thyroid diseases (AIT), indicating a prevalence of 15.5% for thyroid autoimmunity among children diagnosed with T1D. These authors have documented a significantly higher occurrence in girls as opposed to boys, with rates of 21.9% versus 9.3%, respectively (Severinski et al., 2009).

According to study of Ridha et al., for Hashimoto’s thyroiditis, the prevalence is 14%–28% (Ridha and Al Zubaidi, 2019).

In the presented study, patients with and without diagnosed Hashimoto’s disease did not differ significantly in terms of age (38.9 vs. 37.3 years) and diabetes duration (23.2 vs. 22.1 years). Also, mean age at diagnosis (15.5 vs. 15 years) and BMI (25.78 vs. 25.77 kg/m^2^) were similar. Much the same results were obtained from other studies, indicating that among patients with both DM1 and AIT, 62% were female. Additionally, individuals with positive thyroid peroxidase antibodies (anti-TPO) and anti-TG antibodies (anti-thyroglobulin antibodies) were older by 1.95 years and had a slightly longer duration of DM1, only by 1.64 years (Korzeniowska et al., 2015).

This suggests that both autoimmune diseases may occur simultaneously and that female gender is a risk factor. It may be explained by the observation that T1DM patients with positive antibodies to glutamic acid decarboxylase (anti-GAD) are significantly more likely to have anti-TPO and anti-TG (Witek et al., 2012; Hansen et al., 2015).

Understanding the etiopathogenesis of DM1 helps shed light on its common occurrence alongside other autoimmune conditions. Many studies delving into its genetic basis and molecular mechanisms primarily concentrate on the MHC locus responsible for encoding HLA proteins in humans. These genes stand out as pivotal factors contributing to the coexistence of AIT and DM1 (Kahles et al., 2015).

AIT is characterized *inter alia* by the presence of thyroid-specific antithyroid peroxidase (TPO-Ab). A normal range of TPO-Ab (+) was defined as below 16 IU/mL (LSI Medience Corporation Information, 2017).

In our study, the average TPO-Ab (+) was 72.3 IU/mL (Mdn 13.8; ranges 4–610 IU/mL). Among female was 114.9 IU/mL (Mdn 25.6; ranges 5–610 IU/mL), among male 42.5 IU/mL (Mdn 11.5; ranges 4–463.8 lU/mL). Moreover, logistic regression revealed that chances of Hashimoto’ disease among diabetics occurrence increased by 0.6% with each unit increase in TPO-Ab (OR 1.006, 95% Cl: 1.003–1.008; p < 0.05).

In most cases, the course of Hashimoto’s disease in children with diabetes is asymptomatic, and therefore the use of screening tests immediately after the diagnosis of diabetes and their systematic repetition during the course of diabetes ensures early detection of thyroid dysfunction, which can prevent the development of overt hypothyroidism of this gland, with all its consequences.

Among 306 patients with TDM1, retinopathy was found in 54 patients (17.6%). The gender distribution were similar, with women accounting for 44.4% (n = 24) and men for 55.6% (n = 30) of all patients with retinopathy. The presented research demonstrated a statistically significant relationship between the duration of diabetes and the occurrence of retinopathy. Retinopathy appears to be significantly more prevalent among individuals with diabetes of longer duration (p < 0.05). The data suggests a clear association between age and duration of diabetes as shared risk factors for retinopathy. Notably, individuals with retinopathy had an average age of 43.4 years compared to 36.4 years for those without retinopathy. Similarly, the group without retinopathy had a mean diabetes duration of 21.1 years, while those with retinopathy had a longer average duration of 28.2 years. These findings strongly imply that both age and prolonged diabetes duration contribute significantly to the likelihood of developing retinopathy.

The results of multivariate logistic regression indicated that the coexistence of hypertension more than doubles the chance of developing retinopathy (OR 2.096, 95% Cl: 1.035–4.248, p = 0.040). Moreover, the chance of disease occurrence increased by 4.5% with each year of a patient’s age compared to the previous year (OR 1.045, 95% Cl:1.011–1.080, p = 0.009) and the chance of disease occurrence raised by 12.8% with each year of diabetes duration compared to the previous year (OR 1.128, 95% Cl: 1.076–1.182, p < 0.05).

According to the data from IDF, retinopathy in Poland affects 2.8% of patients with type 1 diabetes (International Diabetes Federation, 2023b). Our results are supported by findings from other studies. Bulum et al.'s research in T1DM demonstrated a significant association among diabetic retinopathy (DR), systolic blood pressure (p = 0.035), median levels of HbA1c (p < 0.05), and hypertensive retinopathy (p < 0.05) (Bulum et al., 2022). Another study reported that among diabetic individuals, those with hypertension had a higher proportion reporting diabetes neuropathy and retinopathy compared to diabetic individuals without hypertension (Kesavamoorthy et al., 2015).

The development of DR is influenced by various factors, such as the duration of diabetes, high blood sugar levels, hypertension, abnormal lipid levels, and pre- pregnancy diabetes (Axer-Siegel et al., 1996; Yao et al., 2021). As studies show, hypertension is one of the most common complications of diabetes. In the provided study, nearly every third (28.8%) of the participants had hypertension (13.5% women; 39.4% men). In accordance with findings from the 3B study conducted in China, the prevalence of hypertension among individuals with diabetes was reported to be 59.9% (Zhang et al., 2019). Another study revealed that 75.6% of diabetic patients were affected by hypertension (Ma et al., 2022).

Among 306 patients with type 1 diabetes, neuropathy was found in 25 people (8.2%). The gender distribution was similar. Among patients with neuropathy, women accounted for 52% (n = 13) and men for 48% (n = 12).

Logistic regression revealed that the chance of disease occurrence increased by 10.8% with each year since diabetes diagnosis compared to the previous year (OR 1.108, 95% Cl:1.062–1.156, p < 0.05) and the chance of disease occurrence raised by 17.6% with each year of diabetes duration compared to the previous year (OR = 1.176, 95% Cl:1.0992–1.267, p < 0.05).

According to the data from IDF, neuropathy in Poland affects 4.9% of patients with type 1 diabetes (International Diabetes Federation, 2023a). Feldman et al. described that about half of individuals diagnosed with either type 1 or type 2 DM experience diabetic neuropathy at some point in their lifetime (Feldman et al., 2019).

The prevalence of diabetic neuropathy (DN) among patients with T1DM range from 8% to 54%. This variation could be due to *inter alia* differences in causes methodologies or diagnostic criteria between studies (Martin et al., 2014; Hicks and Selvin, 2019). A systematic review and meta-analysis of subjects with T1DM found a higher pooled prevalence of peripheral neuropathy in patients aged above 16 years than those under 16 years (59.1% vs. 9.5%) and in those with diabetes duration >10 years compared to disease duration <10 years (35.0% vs. 9.4%) (Abdel-Motal et al., 2017).

Similarly, in the present study, patients with neuropathy were significantly older than those without (mean ages 51.3 vs. 36.4 years). In addition, those with neuropathy had a longer duration of diabetes (26.6 vs. 21.9 years) and an older age at diabetes diagnosis compared to those without (24.7 vs. 14.3 years).

Approximately half of patients with T1DM who develop neuropathy may not exhibit any symptoms; indeed, DN is often underdiagnosed, not just by general physicians but also by specialists in endocrinology. This underscores the significance of routinely screening T1DM patients for DN. It is recommended to commence screening for DN five years following the diagnosis of T1DM and to repeat these screenings at least once a year thereafter (Nkonge et al., 2023). A systematic review of 17 randomized controlled trials found that intensive glucose control led to a substantial reduction in the risk of clinical neuropathy (1.84%/year risk reduction [95%CI: 1.11, 2.56], p = 0.00001) and improved nerve function and vibration perception threshold among patients with T1DM (Callaghan et al., 2012).

In the present study, mean BMI was 25.77 kg/m^2^ (26.13 for men; 25.25 kg/m^2^ for women) and ranged from 17.71 to 41.36 kg/m^2^ (18.08–41.36 for men; 17.71–36.25 kg/m^2^ for women). Univariate correlation found that diabetes duration was positively correlated with BMI (p = 0.045).

Although numerous studies have found - higher BMI to be a risk factor for retinopathy, neuropathy and CVD (De Block et al., 2005), no such correlation was detected in the presented study. Nevertheless, BMI was positively correlated with lipid profile parameters, suggesting that it may have an influence on the development of cardiovascular disease.

A growing body of epidemiological evidence indicates that type 1 diabetes is associated with the onset of obesity. This may be due to the impact of a prevailing obesogenic environment or the unique biopsychosocial burden associated with T1DM. Furthermore, intensive insulin therapy, the primary treatment for T1D, may also encourage weight gain, due to a complex interaction between physiological changes associated with exogenous insulin (such as imbalances in insulin distribution and calorie retention) and psychological adjustments to prevent hypoglycemia (Kueh et al., 2024). Wasyl-Nawrot et al. report that 91.6% of newly diagnosed cases of autoimmune diabetes in childhood exhibited normal weight, suggesting no correlation between the rising incidence of T1DM in the pediatric population and higher BMI (Wasyl-Nawrot et al., 2020).

In the present study, univariate correlation analysis demonstrated a positive association between BMI and CRP level (p < 0.05). Hence, BMI appears to be the primary factor influencing CRP. This results emphasis the importance of prioritizing obesity prevention in the ongoing care of children with type 1 diabetes.

In individuals with T1DM, CRP level is believed to play an important role in inflammation and the development of micro- and macrovascular complications. Nowak et al. report a substantial increase in CRP levels among diabetic patients with diabetic retinopathy compared to those without suggesting a connection between inflammation and the emergence of diabetes-related microvascular issues (Nowak et al., 2010).

In the present study no significant differences in CRP levels were found between patients with diabetic retinopathy or neuropathy and those without.

Although there is limited data on elevated CRP levels in diabetic patients with neuropathy or retinopathy, thus linking inflammation with the emergence of microvascular complications, the CRP ratio could serve as a biochemical indicator for the advancement of retinopathy or neuropathy. The mean CRP level for all patients was 2.8 mg/L (range 0.1–22.8 mg/L). A higher mean CRP level was noted among women (3.4 mg/L; 0.2–15.5 mg/L) than among men (1.2 mg/L; 0.1–22 mg/L).

The influence of sex on CRP concentrations is a controversial topic. Our data indicate that the median CRP level was almost twice as high in women compared with men; this is in line with most other studies which have found women to exhibiting higher plasma CRP levels compared to men. This contrast could stem from differences variations in both visceral and subcutaneous fat levels or in estrogen levels, all of which are known to influence CRP levels (Kwok et al., 2009).

Perez-Segura reports that hsCRP levels are elevated in children with type 1 diabetes compared to BMI-matched non-T1DM control, suggesting that an underlying inflammatory state could increase susceptibility to cardiovascular complications (Pérez-Segura et al., 2020). However, further prospective studies are needed to determine the significance of sex-based differences in CRP levels for predicting CVD risk, and their potential implications in the context of T1DM, where it could influence the development of innovative therapeutic approaches and targets.

In the presented study, the analysis performed on 306 cases, divided into 8 clusters that represent different combinations of the presence or absence of Hashimoto’s disease, retinopathy, and neuropathy, did not show significant co-occurrence of two or three of the aforementioned diseases. The majority of patients (66.3%) do not have any of the three conditions, suggesting that most diabetic patients in this study do not suffer from coexisting diseases like Hashimoto’s disease, retinopathy, or neuropathy. A probable explanation for these results is the fact that the study sample had a relatively young median age (with a median age of 35 years). The largest cluster (50%) was the youngest cluster with an average age of 30 years (25–35 years). The study sample was also characterized by a relatively short duration of T1DM (with a median duration of diabetes of 22 years).

L. Bjerg et al. reported different results due to their older and longer-duration diabetes population. This study included individuals with a median age of 52 years, and those with a higher count of complications were notably older. The median duration of diabetes was 24 years. They observed an almost 3.5-fold difference in diabetes duration between individuals with no complications and those with all three complications: neuropathy, retinopathy, and diabetic kidney disease. In models adjusted for diabetes duration and HbA1c, individuals with neuropathy had an odds ratio (OR) of 2.15 (95% CI: 1.73–2.66) for concurrent diabetic kidney disease. Those with retinopathy had an OR of 2.49 (95% CI: 1.92–3.24) for diabetic kidney disease and an OR of 2.66 (95% CI: 1.94–3.64) for neuropathy. These findings help to better understand the coexistence of these conditions among diabetic patients and can assist in tailoring treatment plans to meet the needs of specific groups (Bjerg et al., 2018).

In summary, diabetes can lead to severe long-term complications. As such diabetes a key focus on preventing vascular disease. To this end, a crucial role in the prediction and prevention of T1DM can be played by technological advances such as screening for autoantibodies, especially in the pediatric years. Indeed, recent years have seen an expansion in screening programs aimed at assessing T1DM risk among in the general population, as well as development of collaborative networks aimed at trialing interventions to delay disease progression and clinical complications; these may include the use of continuous glucose monitoring (CGM) for risk evaluation and tracking blood glucose levels in these environments (Besser et al., 2022).

There were several limitations in this study. Firstly, the sample was drawn exclusively from inpatients at the University Clinical Hospital No. 2 of the Medical University of Lodz, Poland. This regional and somewhat limited sample size may not fully represent the broader population. Additionally, the researchers had to rely on the data available in the patients’ medical records, without the opportunity for verification. This limitation may have led to gaps in the data, particularly concerning risk factors such as smoking and BMI. Furthermore, the absence of longitudinal data on self-management factors—such as physical activity and dietary intake—restricted a more comprehensive analysis of lifestyle influences.

Future research is necessary to validate the clusters and assess their prognostic significance across different populations. The ultimate and most important step will be to determine whether tailoring clinical practice based on patient subgroups leads to better outcomes.

## Conclusion

Patient age, duration of diabetes, and the presence of hypertension are key risk factors for diabetes-related complication. Patient BMI also typically increases over the course of diabetes. Over half of the patients (51.98%, n = 117) had an abnormal BMI, which may increase the risk of diabetes-related complications. BMI was associated with other markers of health status, such as CRP levels, uric acid and FT3. This suggests that controlling body weight may influence the overall health status of diabetic patients. Hypertension is a significant risk factor for many diabetes-related complications, such as retinopathy.

Despite significant advances in the diagnosis and treatment of diabetes, its acute and chronic complications continue to pose a direct threat to patient health and survival. Consequently, the prevention and therapeutic management of these complications remain critical objectives in diabetes care. Given the rising global incidence of diabetes, it is imperative that both healthcare professionals and patients are well-versed in the complications associated with the disease.

## Data Availability

The original contributions presented in the study are included in the article/Supplementary Material, further inquiries can be directed to the corresponding author.

## Supplementary material

SUPPLEMENTARY FIGURE S1 Correlation between BMI vs. CRP.

SUPPLEMENTARY FIGURE S2 Correlation between BMI vs. UA.

SUPPLEMENTARY FIGURE S3 Correlation between BMI vs. FT3.

SUPPLEMENTARY FIGURE S4 Correlation between BMI vs. LDL.

SUPPLEMENTARY FIGURE S5 Correlation between BMI vs. TG.

SUPPLEMENTARY FIGURE S6 Correlation between BMI vs. CHOL.

SUPPLEMENTARY FIGURE S7 Correlation between BMI vs. HDL.

## Abbreviations

ACR, Albumin-to-Creatinine Ratio; AIT, Autoimmune Thyroid Diseases; anti-GAD, Antibodies to Glutamic Acid Decarboxylase; BMI, Body Mass Index; CGM, Continuous Glucose Monitoring; Chol, Cholesterol; CRP, C-Reactive Protein; CREAT, Serum Creatinine; DKD, Diabetic Kidney Disease; DN, Diabetic Neuropathy; DR, Diabetic Retinopathy; FT3, Free Triiodothyronine; FT4, Free Thyroxine; GAD, Glutamic Acid Decarboxylase; GFR, Glomerular Filtration Rate; HA, Hypertension; HbA1c, Hemoglobin A1c; HDL, High-Density Lipoprotein; IA2, Insulinoma-Associated Antigen; IDF, International Diabetes Federation; LDL, Low-Density Lipoprotein; M, The Mean; Mdn, Median; min-max, Minimum-Maximum; PAD, Peripheral Artery Diseases; SD, Standard Deviation; T1DM, Type 1 Diabetes Mellitus; TPO-Ab, Thyroid-Specific Antithyroid Peroxidase; TPO antibodies, Thyroid Peroxidase Antibodies; TSH, Thyroid-Stimulating Hormone; UA, Uric Acid; UACR, Urinary Albumin-to-Creatinine Ratio.
