# Supplementary figures and images for: An analysis of epidemiological characteristics of microvascular complications and comorbidities among type 1 diabetes patients
Source: Acta Biochim Pol. 2025 May 22;72:14569. doi: 10.3389/abp.2025.14569 (PMC12137067; doi:10.3389/abp.2025.14569)

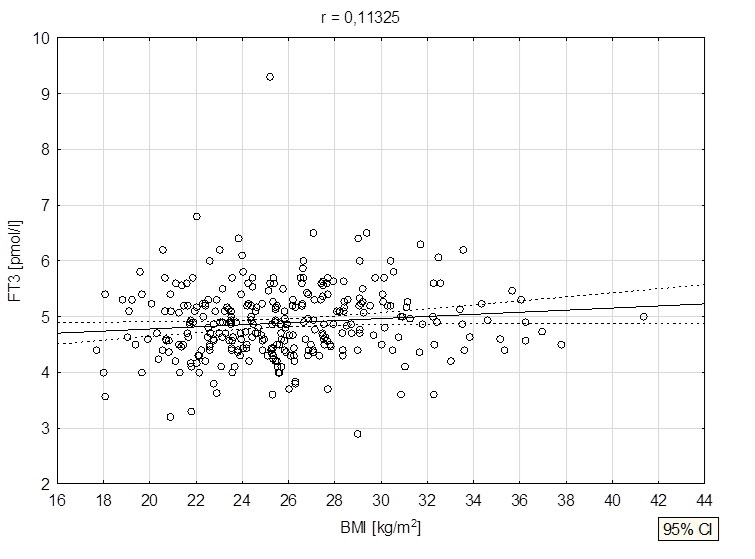

Supplement: Supplementary file 1 [file Image3.JPEG]

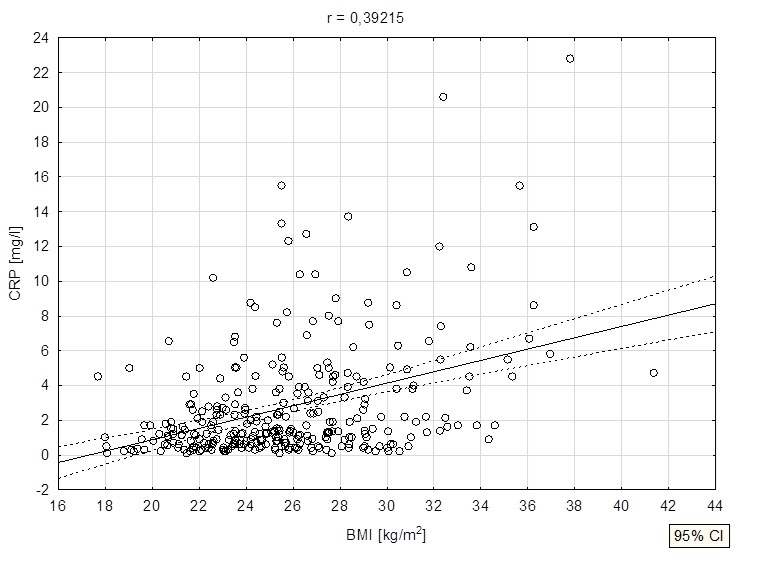

Supplement: Supplementary file 2 [file Image1.JPEG]

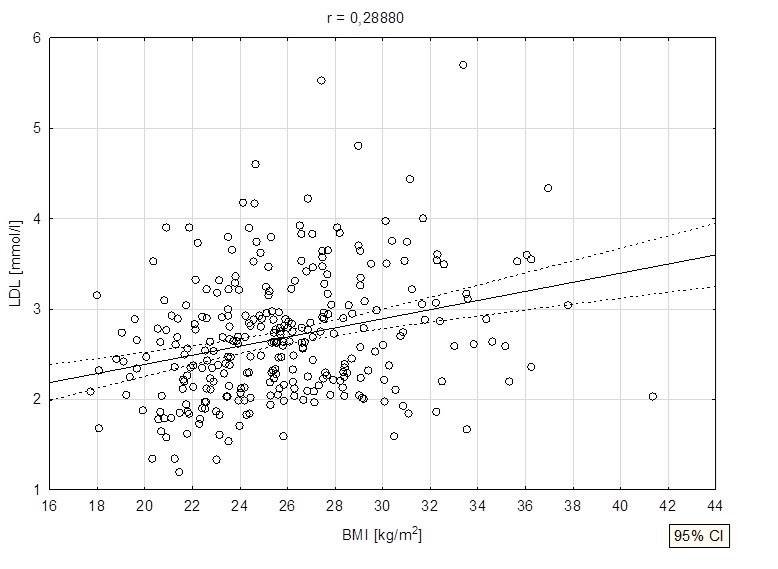

Supplement: Supplementary file 3 [file Image4.JPEG]

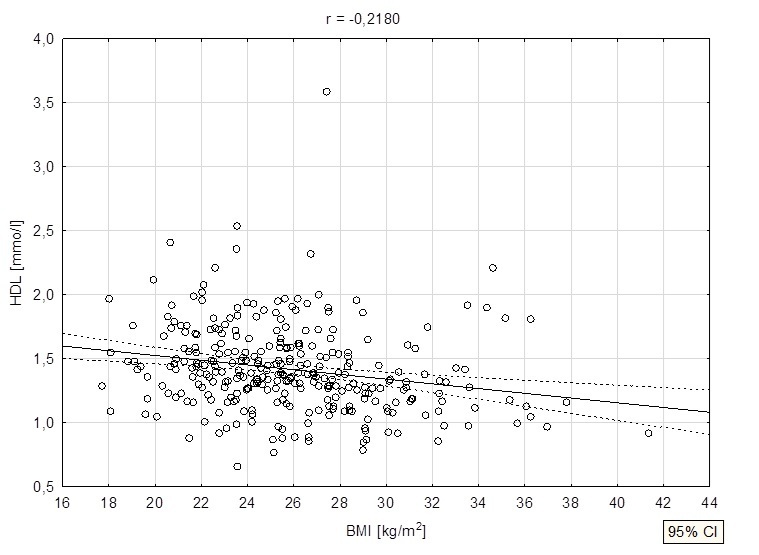

Supplement: Supplementary file 4 [file Image7.JPEG]

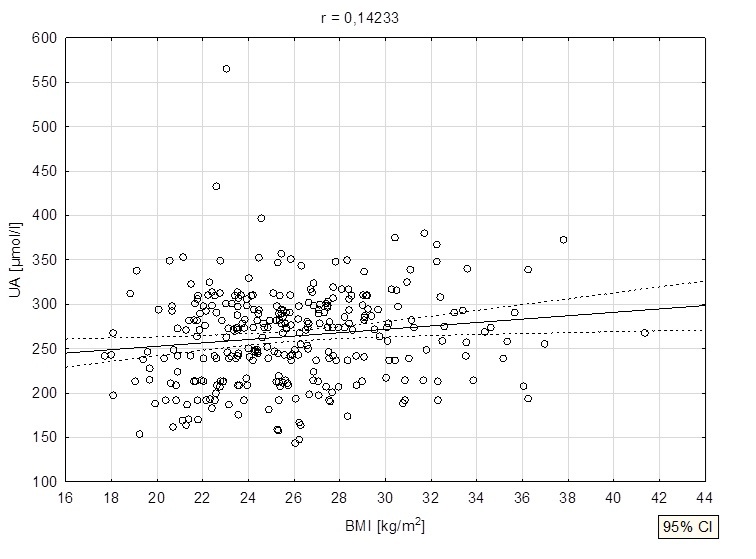

Supplement: Supplementary file 5 [file Image2.JPEG]

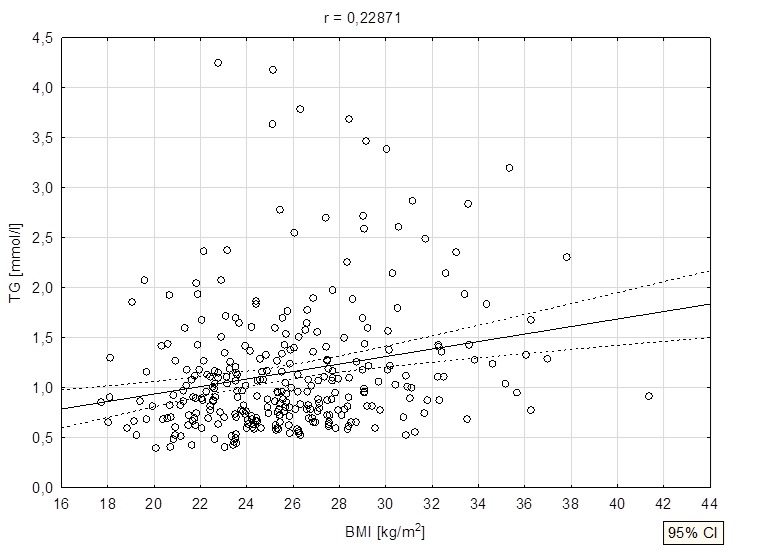

Supplement: Supplementary file 6 [file Image5.JPEG]

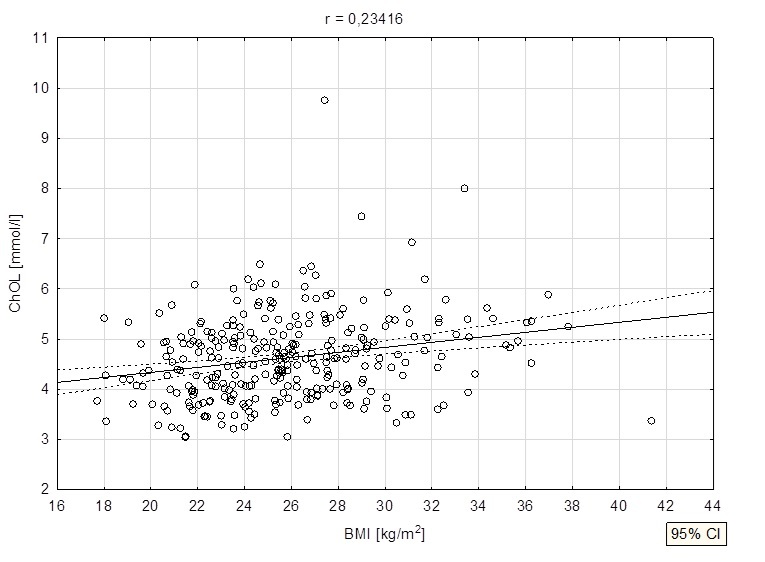

Supplement: Supplementary file 7 [file Image6.JPEG]
